# Hemorrhagic meningioma with pulmonary metastasis: Case report and literature review

**DOI:** 10.1515/biol-2022-0745

**Published:** 2023-10-23

**Authors:** Xuan Cao, Qiaowei He, Mingzeng Ding, Wei Kong, Changyou Yin, Wei Zhao, Yanbin Wang

**Affiliations:** Department of Neurosurgery, The Affiliated Yantai Yuhuangding Hospital of Qingdao University, Yantai, 264000, China; Department of Neurosurgery, People’s Hospital of Rongcheng, The Middle Section of Chengshan Avenue 298, Rongcheng, 264300, Shandong, China

**Keywords:** hemorrhagic meningiomas, metastases, diagnosis, case report

## Abstract

Meningiomas are extra-axial neoplasms that originate from the arachnoid cap cells located on the inner surface of the meninges. Approximately 36% of central nervous system tumors are meningiomas. Based on earlier findings to be benign in most cases, they are categorized as slow-growing tumors that form gradually over time. Meningiomas are usually asymptomatic and discovered inadvertently. They rarely present with immediate clinical symptoms or abrupt hemorrhagic strokes. However, tumor hemorrhage can be fatal in high-grade meningiomas, particularly those with vascularization. We describe a 58-year-old man who was hospitalized after experiencing an unexpectedly acute headache. The right cerebellar hemisphere and vermis cerebellar hemorrhage were detected on computed tomography (CT), and the cerebellar hemorrhage was explained by a diagnosis of hypertension. When additional analysis of the patient’s chest CT indicated lung mass lesions, we assumed that the lung cancer had spread to the brain. However, the pathological outcomes of a guided definite pulmonary aspiration biopsy, in conjunction with resection of the cerebellar tumor, suggested a subtentorial meningioma with ruptured hemorrhage and pulmonary meningioma metastasis. The patient was transferred to a hospital closer to home for ongoing follow-up and, after 2 months, he had recovered well.

## 1 Introduction

More than one-third of primary central nervous system malignancies are meningiomas, which are the most common intracranial tumors [1,2,3]. The pathological types vary, with 20% of cases suggesting malignant histological features corresponding to meningioma grades II or III and higher grades of recurrence and aggressive behavior [4]. Metastatic meningioma is an uncommon clinical disease with an estimated incidence of 0.1–0.76% among all patients with meningioma [5]. Concomitant occurrences are uncommon due to the rarity of tumor hemorrhage, which only occurs in 0.5–2.4% of all documented cases of meningiomas [6].

Herein, we present the case of a middle-aged man who had a sudden headache and was initially diagnosed with hypertensive cerebellar hemorrhage on head computed tomography (CT). However, following additional magnetic resonance imaging (MRI) of the head, and chest CT, the diagnosis changed to lung cancer with brain metastases. A diagnosis of hemorrhagic stroke complicated by posterior anaplastic meningioma World Health Organization (WHO) grade III with pulmonary metastases was confirmed based on the final pathological results.

## 2 Case report

We report the case of a 58-year-old man who was admitted after experiencing a persistent, severe headache for 5 h, and two episodes of vomiting; however, no further symptoms or coffee-like substances were detected. He had a Glasgow Coma Scale (GCS) [7] score of 15, no neurological symptoms, and no history of cancer.

**Figure 1:**
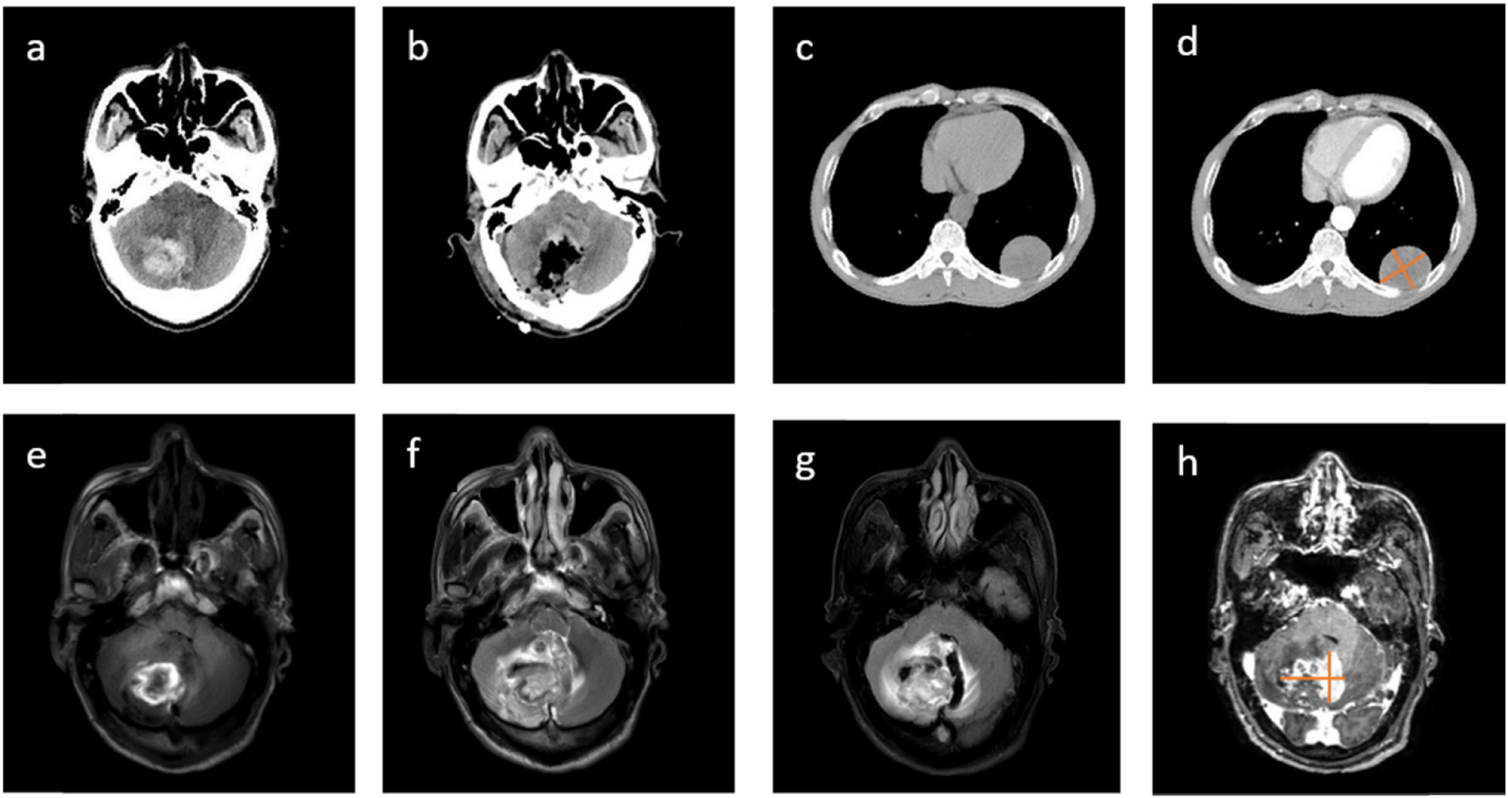
CT scan of the emergency room showed hemorrhage in the right cerebellar hemisphere (a). Postoperative CT scans revealed clear axial plane views of the surgically cleansed meningiomas and hematomas (b). (c) An image of a subpleural mass in the left lower lobe with well-defined, well-intact margins, and a slightly less uniform density. After enhancement scanning, there is a little heterogeneous enhancement of the chest occupation, tumor size is about 3.4 cm × 3.2 cm (d). The right cerebellar hemisphere exhibits a patchy confluence of T1-weighted, T2-weighted, and FLAIR signals (e–g); the peripheral T2-weighted edema signal has fuzzy boundaries. (h) After enhancement scanning, tumor tissue shows a faintly heterogeneous enhancement. The size of the tumor in the image is about 4.3 cm × 2.1 cm.

An emergency department CT scan of the head revealed cerebral hemorrhage in the right cerebellar hemisphere and vermis (Figure 1a). The results of the examination were as follows: the right cerebellar hemisphere slice images with T1-weighted (Figure 1e), T2-weighted (Figure 1f), and FLAIR (Figure 1g) signals were confusing. On the second day of hospitalization, MRI and head examination findings were significantly enhanced. A T1-weighted hyperintense and tiny patch of enhancement was visible in the center of the enhanced dissected lesion, while slightly longer T2-weighted edema with blurred boundaries was visible at the periphery of the lesion (Figure 1h). Right cerebellar MRI revealed a tumor with hemorrhage as an aberrant signal. Chest CT of the left subpleural lobe revealed a well-defined subpleural mass with somewhat less uniform density and slightly less uniform enhancement following contrast-enhanced CT (Figure 1c and d). After discussions with radiologists and neurosurgeons, it was decided that lung-occurring brain metastases were more probable.

The patient underwent right hemisphere tumor resection and external ventricular drainage because of a headache that progressively worsened throughout hospitalization, and a change in consciousness (from lucid to lethargic). We first inserted a right frontal horn external ventricular drain, which allowed us to reduce intracranial pressure during surgery by releasing cerebrospinal fluid and expanding the surgical space, taking into consideration the patient’s gradual increase in intracranial pressure. This prevented the brain tissue from swelling after the skull was opened surgically as well as the development of severe swelling during surgery. Intraoperatively, the tumor appeared fleshy red, mushy, and hypervascular, with peritumoral hematomas. The tumor’s excision size was approximately 4.5 cm × 3.2 cm × 2.5 cm, and there was no clear separation between the tumor and the surrounding brain tissue. Although it was not clear whether the tumor was a high-grade meningioma during the operation, the fact that the tumor was hemorrhagic made us more cautious; we excised the dura that was adherent to the tumor and used an artificial dura to reduce the tension and suture it. We considered that the patient was hemorrhagic from the tumor, and decompression was performed using a debulking flap. Postoperative CT of the head was performed (Figure 1d).

**Figure 2:**
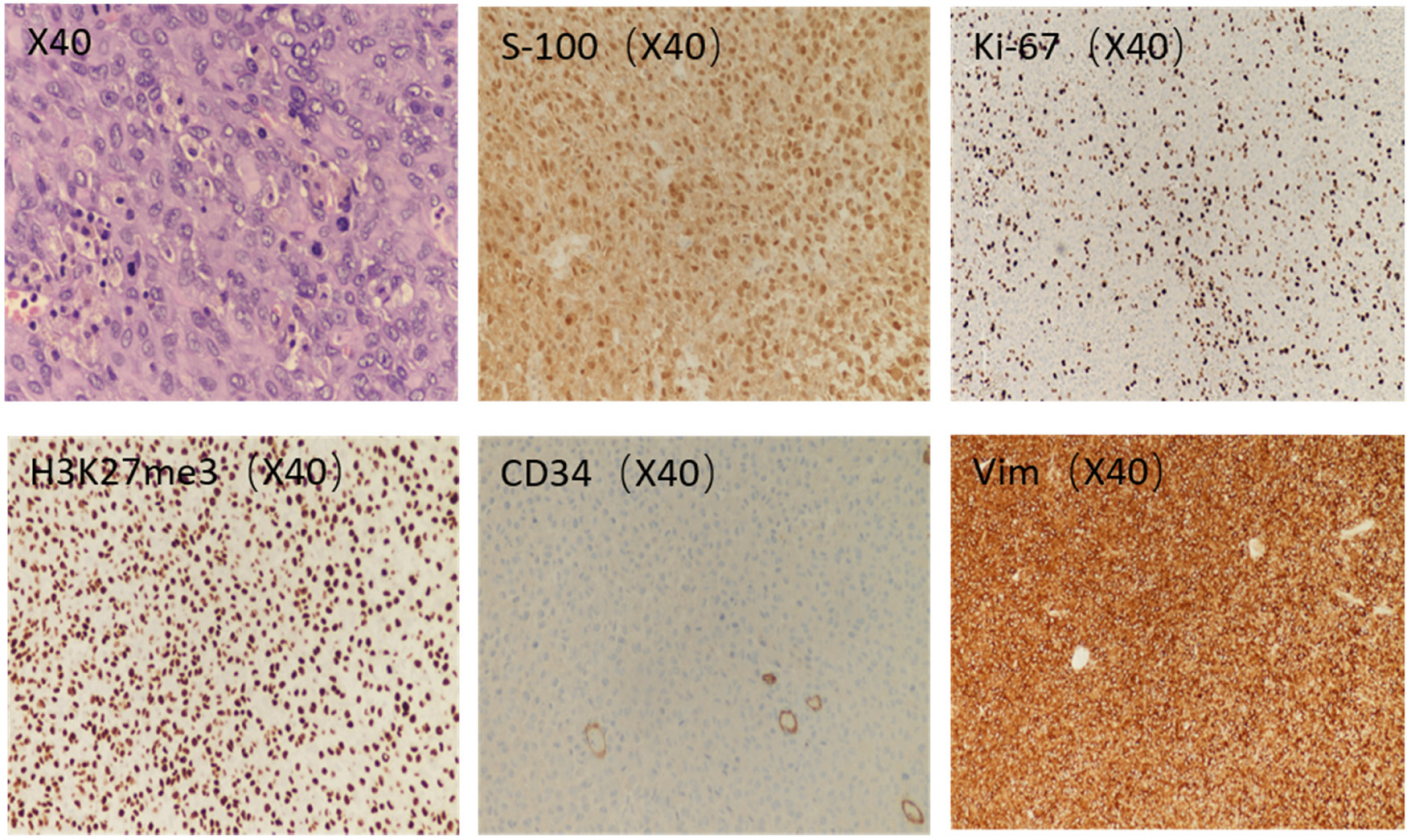
Neuropathological features of anaplastic meningioma. Malignant mesenchymal brain tumor, marked cell atypia, and prominent mitotic label (>20/10HFP). HE (×40). The cytoplasmic S-100 immunoreactivity was positive. Ki-67 labeling index shows nuclear immunoreactivity. H3K27me3 and CD34, which are both positively expressed in tumor tissue by immunohistochemistry, were found. Cords of tumor cells with intense Vim immunoreactivity.

A malignant mesenchymal brain tumor (right cerebellar mass) was resected, with extremely diverse, and markedly mitotic cells (>20/10 HPF). The immunohistochemical findings were as follows: s-100 (+), a 60% positivity rate of KI-67, H3K27ME3 (+), CD34 (+), Vim (+) and STAT-6(−); the postoperative pathological findings are shown in Figure 2. The lesion was classified as an anaplastic meningioma (WHO III).

**Figure 3:**
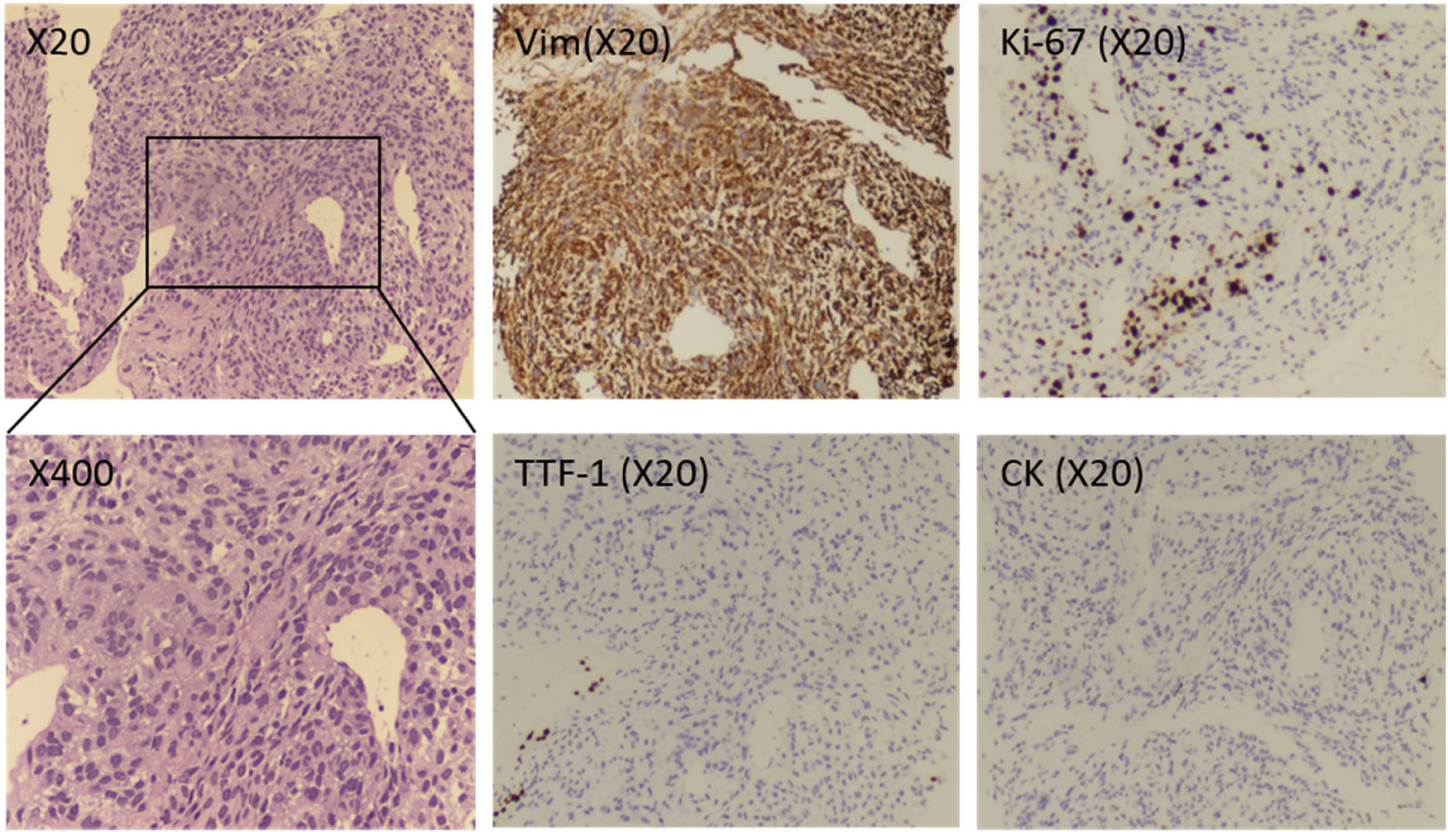
Histopathological features of the needle biopsy of the left lung tumor. HE staining revealed that the tumor tissue does not invade lung tissue (×20), the presence of mitotic segments and microscopic heterogeneity are both obvious (×400). The ki-67 positivity rate is >30%. Tumor cell cords with potent Vim immunoreactivity. Immunohistochemistry staining showed that the tumor was negative for TTF-1 and CK, respectively.


When comparing the pathology of head and lung cancers, meningioma metastases to the lung should be considered for left lung tumors identified using needle biopsy (Figure 3): Vim(+), Ki-67 positivity 48%, TTF1(−), and CK(−). One week after treatment, the patient was transferred to a hospital closer to home for ongoing rehabilitation, and he underwent postoperative CT of the head (Figure 1b). Two months later, a telephonic checkup revealed that the patient had recovered well.

**Informed consent:** Informed consent has been obtained from all individuals included in this study.
**Ethical approval:** The research related to human use complied with all the relevant national regulations, and institutional policies and in accordance with the tenets of the Helsinki Declaration, and has been approved by the Medical Ethics Committee of Yantai Yuhuangding Hospital (approval number: 2023–253).

## 3 Discussion

### 3.1 Clinical characteristics

The second highest incidence of meningiomas, predominantly composed of arachnoid cells and struts, is observed in the nervous system. Convex and cranial base subarachnoids can arise from various embryonic layers, which explains why meningioma development rates vary from one area of the brain to another. Most studies have reported that intracranial meningiomas are prone to exponential growth [8,9,10,11], and they arise 2.8 times more frequently in women [12]; however, men tend to develop anaplastic meningiomas more frequently [13]. However, most meningiomas grow at a linear pace of 2–4 mm/year [14].

Meningiomas typically originate on the convex surface of the brain (adjacent to the sagittal sinus, falx, or venous sinus) and thereafter in the sphenoid spine, olfactory groove, stellar region, and posterior cranial fossa at the base of the skull. Epidural and ventricular lesions are rare. Convex surfaces and other non-basal regions have the highest incidence of high-grade meningiomas. Approximately 3–6% of intracranial meningiomas are tentorial [15,16]. The WHO 2016 classification divides meningiomas into three grades, WHO grade I (benign), grade II (intermediate), and grade III (malignant) [4], containing 15 different meningioma subtypes. About 80% of WHO grade II and grade I and grade II up to 20% may display aggressive behavior [17,18,19,20].

### 3.2 Radiological features

MRI and additional CT are usually used to diagnose meningiomas. However, depending on the pathogenic type, meningioma signals on MRI vary. WHO grade I tumors and T2-weighted signals have different associations; grade I tumors are not related to T1-weighted signals. Invasive meningiomas are also more likely to exhibit T2WI hyperintensities [21], whereas meningiomas with calcification and T2WI hypointensities or similar on MRI have lower growth potential [22,23].

Diagnostic discrimination can be performed in conjunction with diffusion-weighted imaging, magnetic resonance spectrum, and perfusion-weighted imaging for atypical meningiomas [24,25,26,27]. In recent years, there has been a gradual increase in the radiomics of meningiomas, combining imaging features of different grades of meningiomas with the pathological and molecular features of the tumors. It has great predictive power for tumor pathology grading, subtypes, recurrence, differential diagnosis, and brain tissue invasion [3]. Additionally, the use of the mean apparent diffusion coefficient value (ADCmean) and conventional MRI characteristics as noninvasive preoperative diagnostic tools for anaplastic meningiomas may have therapeutic benefits [23]. However, factors with strong predictive values for higher tumor grades have been studied further at multiple centers.

### 3.3 Meningioma metastasis

WHO grade II and III meningiomas are more likely to show recurrence, aggressive behavior, and distant metastasis [3]. With an estimated incidence of 0.1–0.76% among all meningioma patients, metastatic dissemination of meningiomas is a rare clinical complication. Regarding the tumor grading, there are no discernible differences in the sizes of the detected metastases [28].

A total of 115 patients who had 164 metastases of metastatic meningiomas between 1990 and 2012 were reviewed; approximately 33.9% of the cases were considered to be grade I, 20.9% were considered to be grade II, and 40% were found to be grade III. The most common sites of metastatic disease were the lungs (37.2%), bones (16.5%), intraspinal areas (15.2%), and the liver (9.2%) [29]. Meningioma metastasis is associated with WHO grade III meningiomas. In one series, the incidence of metastasis was 0.67% for all meningiomas and was higher in WHO grade II (2%) and III (9%) meningiomas, which is consistent with the metastatic trend reported by most clinical investigators [20,30]. The literature suggests that tumor invasion of the venous sinus system, ventricular system, or skull is common with metastatic meningiomas. Tumor infiltration of the venous sinuses occurs mainly in the superior sagittal, cavernous, occipital, and transverse sinuses. Nearly all distant metastases of meningiomas infiltrating the venous sinus system occur in the lung [31]. Although meningiomas possess considerable metastatic potential, many researchers are intrigued by the very low overall rate of metastasis. Simply put, the tight cohesion of tumor cells, the lack of cerebral lymphatics, and the probable absence of “fertile soil” at distant locales may all contribute to the rarity of extracranial metastases [32].

### 3.4 Intracranial hemorrhage from a meningioma

Approximately 1.5–5.4% of all tumors, especially in malignant pathologies such as metastatic neoplasms or gliomas, occur with intracranial hemorrhage [33]. Spontaneous bleeding from meningiomas is rare. Documented HMs account for only 0.5–2.4% of cases [6]. Despite the appearance of multiple exhaustive reviews, numerous uncertainties still exist surrounding the pathophysiological mechanism associated with hemorrhage from an unsuspected meningioma.

These reviews have led to the following current hypotheses regarding hemorrhage [34,35,36,37]: (1) the artery that supplies the tumor grows tortuous as the tumor grows, the vessel walls become more brittle and less elastic, and the ability to control stress reactions like blood pressure variations are lost, leading to hemorrhage; (2) vascular networks that are growing abnormally or are immature within meningiomas under specific circumstances may result in hemorrhage; (3) bleeding may be a result of a meningioma with distensive growth or from lacerations of the pontine veins; (4) hemorrhage may occur owing to burst intratumor or peritumoral veins, followed by increased intratumor pressure and venous regurgitation as a result of thrombosis or thrombosis of the vessels in the meningioma; and (5) hemorrhage may also be brought on by other systemic causes that are not directly linked to the tumor, such as coagulation abnormalities, peritumoral edema, and tumor malignancy. In a large-scale study, the following factors were identified as risk factors for meningioma bleeding: (1) age of 30 years or >70 years; (2) tumor location in the ventricle or convex meningioma; (3) highly malignant, fibrous, or angioblast-producing lesions on histological examination; and (4) hypertension, anticoagulation, and traumatic brain injury [38]. Based on this, Pressman et al. [36] hypothesized that high-dose estrogen replacement and serotonin modulation therapy may be unique causes of meningioma hemorrhage.

### 3.5 Management and prognosis

We report the case of a 58-year-old man with postoperative pathology confirmed as WHO grade III meningioma complicated by hemorrhage without a supratentorial tumor. The patient’s head MRI results were suggestive of a tumor hemorrhage; we considered metastases and hemangioblastomas for tumors in the posterior cranial fossa and did not consider high-grade meningiomas. In general, meningiomas can be diagnosed using clinical features together with specific MRI findings. However, the probability of meningioma hemorrhage is low, and subsequent concave meningioma hemorrhages are much less frequent. Therefore, we usually treat meningiomas as benign tumors and ignore the meningioma diagnosis.

Heavy bleeding and the absence of typical imaging characteristics are more likely to be inappropriately identified as acute cerebrovascular ailments in individuals with HMs who have undergone insufficient imaging examinations [39]. Our patient was admitted to the hospital in an emergency with a simple diagnosis of cerebral hemorrhage.

The characteristics of meningiomas and the side effects of different treatments for different patients vary widely, necessitating individualized therapy. However, the Simpson grade [40] is still used as a measure of the extent of resection for growing and symptomatic meningiomas of different grades. Radiotherapy and stereotactic radiosurgery are used as adjuvant treatments for newly diagnosed atypical and degenerative meningiomas, especially in cases where complete resection is not possible.

Adjuvant external beam radiation therapy (EBRT) is very commonly used to treat WHO grade III meningiomas; however, there are no biomarkers to predict the response to therapy, and there is a limited understanding of how to optimize adjuvant EBRT for patients with WHO grade III meningiomas. One week after surgery, the patient was transferred to a rehabilitation hospital. We recommend that the patient undergo radiotherapy at our center as soon as possible; however, the patient chose to undergo radiotherapy at a medical center closer to his home, based on factors such as accessibility and health insurance.

If a meningioma metastasizes and recurs, the prognosis is poor, and the patient’s chance of survival is significantly reduced [41,42]. Local therapeutic alternatives, including surgery and radiotherapy, continue to pose a therapeutic challenge for patients with metastatic and recurring meningiomas. Many researchers and doctors continue to attempt to treat meningiomas systematically, but there are currently scant historical benchmark data on the outcomes of high-grade meningiomas and controlled trials [40]. Systemic therapeutic techniques being developed in meningioma treatment research include anti-angiogenic medications, immunotherapeutic approaches, and, more recently, genomic studies.

## 4 Conclusion

Meningiomas are often asymptomatic and any primary symptoms (focal neurological symptoms, seizures, and increased intracranial pressure) may often be linked to the effects of a mass. Although meningiomas are typically benign, meningioma hemorrhage and metastatic cases have been documented. Meningiomas may only rarely arise within the posterior fossa; nonetheless, they usually develop along the falx, sphenoid bone, and where there is convexity. Regular follow-up is crucial because of the likelihood of recurrence after meningioma surgery, even if the tumor has been completely resected.

## Supplementary Material

Supplementary Table
