# Supplementary material for: Hemorrhagic meningioma with pulmonary metastasis: Case report and literature review
Source: Open Life Sci. 2023 Oct 23;18(1):20220745. doi: 10.1515/biol-2022-0745 (PMC10628577; doi:10.1515/biol-2022-0745)
Supplement: Supplementary Table [file biol-2022-0745-sm.pdf]

Supplementary material

Table S1: Glasgow Coma Scale

Eye opening (E)	Verbal response (V)	Best motor response (M)
1 None	1 None	1 None
2 To pressure	2 sounds	2 Extension
3 To speech	3 Words	3 Abnormal flexion
4 Spontaneous	4 Confused	4 Normal flexion(withdrawal)
	5 Orientated	5 Localising
		6 Obeying commands

Each component is assessed by a standerdised approach that permits objective evaluation and documentation of information about the level of consciousness.

Table S2: Molecular characteristics of meningioma subtypes in WHO classification 2021

Items	Subtypes	Common mutations	CNVs
WHO grade 1	Meningothelial	*AKT1(/TRAF7),SMO*	del 22q
	Fibroblastic	*NF2*	del 22q
	Transitional	*NF2*	del 22q
	Secretory	*KLF4/TRAF7**	Unknown
	Psammonmatous	*NF2*	del 22q
	Metaplastic	*NF2*	gain 5
	Microcystic	*NF2*	gain 5
	Angiomatous	*NF2*	gain 5
	Lymphoplasmacyte-rich	Unknown	Unknown
WHO grade 2	Atypical	*NF2*	del 1p, del 22q, del 14q
	Chordoid	None	Del 2p
	Clear cell	*SMARCE1*	None
WHO grade 3	Anaplastic	*NF2,TERT* promoter*	Del 1p, 10, 14, 22q, homo del *CDKN2A/B**
2016	Rhabdoid	*BAP1*	*BAP1* locus
WHO grade 3	Papillary	*PBRM1*	No specific

*Novel molecular criterion for subtypes, besides histology features, in WHO classification 2021. CNVs: Copy number variations; del: Deletion; homo del: Homozygous deletion;.

Table S3: The Simpson Grade

Grade	Definition	Symptomatic recurrence at 10 years
I	Complete removal including resection of the underlying bone and associated dura	9%
II	Complete removal and coagulation of dural attachment	19%
III	Complete removal without resection of dura or coagulation	29%
IV	Subtotal resection	44%
V	Simple decompression with or wihtout biopsy	11%
